# The Combined Effect of Exercise and Behavioral Therapy for Depression and Anxiety: Systematic Review and Meta-Analysis

**DOI:** 10.3390/bs10070116

**Published:** 2020-07-14

**Authors:** Kelsey Bourbeau, Terence Moriarty, Akeisha Ayanniyi, Micah Zuhl

**Affiliations:** 1 Department of Health, Exercise, and Sports Sciences, University of New Mexico, Albuquerque, NM 87131, USA; akeisha@unm.edu; 2 Department of Kinesiology, University of Northern Iowa, Cedar Falls, IA 50614, USA; terence.moriarty@uni.edu; 3 School of Health Sciences, Central Michigan University, Mount Pleasant, MI 48859, USA

**Keywords:** depression, anxiety, exercise, behavioral therapy, CBT, exercise intensity, mental health

## Abstract

Behavioral therapy (BT) and exercise are efficacious treatments for depression and anxiety when employed separately. The combination of BT and exercise (BT+Ex) may augment improvements but the combined effect of these therapies is not fully elucidated. The purpose of this meta-analysis was to determine if BT+Ex yielded a significant reduction in depression and anxiety symptoms compared to BT alone (BT). Randomized controlled studies published prior to September 2019 were searched among several databases (PUBMED, MEDLINE, PsychArticle, and Cochrane Central Register of Clinical Trials). Studies that measured depression and anxiety symptoms following BT+Ex vs. BT were extracted and analyzed. The effect of these therapies on depression and anxiety were analyzed. Subgroup analyses were performed to evaluate the effect of exercise intensity (moderate and high), exercise type (aerobic and combined exercise), and baseline levels of depression. The moderating effects of gender, age, and treatment duration were performed. Data were extracted from 18 studies (1686 participants, mean age = 47 years, 65% female). There was a significant effect of BT+Ex on symptoms of depression. The effect of BT+Ex was significant for moderate intensity exercise and elevated baseline levels of depression. Age moderated the effect for depression. There was a significant effect of BT+Ex on depressive symptoms in humans. Exercise intensity and elevated depressive symptoms may play a role in the effect of exercise.

## 1. Introduction

The World Health Organization estimates that globally, 322 million individuals suffer from depression and 264 million individuals suffer from anxiety [1]. Depressive disorders are ranked as the single largest contributor to non-fatal health loss and are also linked to an estimated 14.3% of deaths worldwide [2]. In addition, depression and anxiety occur comorbidly with several diseases (e.g., cardiovascular, respiratory, neurological, and metabolic) [3] and increase the risk of cardiovascular disease [4,5], type 2 diabetes mellitus [6,7], and hypertension [8,9]. Further, comorbid depression and anxiety have been shown to predict poor disease outcomes [10,11].

Behavioral therapy (BT) (i.e., cognitive behavioral therapy, interpersonal therapy, counselling) is a common treatment for anxiety and depression [12,13]. Despite widespread use, not all individuals respond to this type of treatment [14,15]. Findings from a review by Hunot et al. [16] suggest that only 46% of individuals with generalized anxiety disorder (GAD) showed a positive response to BT [16]. As a result, clinicians commonly use combined therapies such as multiple forms of BT and/or medication; however, these combination therapies may not provide long-term mitigation of anxiety and depression [17].

Physical exercise has emerged as an efficacious treatment for symptoms of anxiety and depression [18,19]. While the majority of these studies have been done to compare the effectiveness of BT to exercise interventions, fewer studies have been employed to examine the combined effect of exercise and BT (BT+Ex). Researchers have hypothesized that BT+Ex may augment the effectiveness of treatment [20]. Ernst et al. [21] hypothesized that the exercise-induced upregulation of brain-derived neurotrophic factor (BDNF) promotes brain neurogenesis, resulting in a reduction of symptoms associated with depression and anxiety [21]. Physical exercise also improves cognitive functioning (e.g., processing speed, memory, learning), which would support the retention of skills learned in BT [22,23]. In addition, improved self-efficacy and mood state as a result of exercise may also improve BT treatment outcomes. Thus, the combination of exercise with BT may enhance symptom reduction through neurophysiological, cognitive, and improved self-worth mechanisms. It is important to note that the purported mechanisms that explain the benefits of exercise appear to be influenced by components of the exercise intervention, such as type of exercise (aerobic, resistance), duration of each session, and intensity.

A recent meta-analysis examined the effect of exercise combined with BT on depression and anxiety [20]. However, the research group focused on the adjunct effect of cognitive behavioral therapy (CBT) thereby excluding other forms of behavioral therapy [20]. To date, we are unaware of a previous meta-analysis that has systematically examined the adjunct effect of exercise when combined with various types of BT. Thus, the purpose of this systematic review and meta-analysis is (1) summarizing the literature on the effects of BT+Ex compared to BT alone on depression and anxiety, and (2) identifying potential moderators (exercise programming variables) that may influence the effect of adjunctive exercise. Bernard et al. [20] recently analyzed the effect of exercise combined with cognitive behavioral therapy (but not other types of BT) on various symptoms among adults with chronic illness. Several types of BT treatment are used to treat symptoms of depression and anxiety; therefore, the current aim is to provide a more thorough review of the effects of BT+Ex.

## 2. Materials and Methods

### 2.1. Search Strategy

A systematic literature review was performed following the Preferred Reporting Items for Systematic Reviews and Meta-Analyses (PRISMA) guidelines [24]. Searches were conducted in September 2019, thus identifying articles published prior to this date. No restrictions were placed on how long ago the study was published. Potential studies were identified using electronic databases including MEDLINE, PUBMED, PsychArticles, and the Cochrane Central Register of Clinical Trials. Search terms included “depression”, “anxiety”, “behavioral therapy”, “cognitive behavioral therapy”, “physical activity”, and “exercise” in various combinations along with movement-based meditation activities such as “yoga” and “tai-chi”. An example search query included “depression” OR “anxiety” AND “exercise” OR “physical activity” AND “behavioral therapy” OR “cognitive behavioral therapy” AND “randomized controlled trial”. In addition, reference lists of original and review articles were manually evaluated for studies not identified during the database search. After an initial screening of titles and abstracts, studies were assessed for inclusion and exclusion criteria.

### 2.2. Selection Criteria

Included in this meta-analysis were randomized controlled trials (RCT) that (1) enrolled human subjects that were evaluated for depression or anxiety or elevated symptoms of depression or anxiety (by any established criteria or validated screening measure), (2) enrolled subjects aged 18 and above, or (3) compared the effects of behavioral therapy (BT) to the effects of behavioral therapy treatment combined with exercise (BT+Ex) (i.e., aerobic, resistance, mind-body). Studies were excluded if (1) they compared acute treatments (single session of exercise or traditional treatment), (2) the intervention (traditional treatment or exercise) was only educational (i.e., teaching behavioral strategies or how to exercise), or (3) they were cross-sectional or observational in nature.

### 2.3. Data Extraction and Outcome Assessment

Data extraction was carried out independently by two authors and disagreements were resolved by consensus. The following information was extracted from each article: Subject characteristics (sex, age, race, disease condition), study characteristics (publication year, country of origin, intervention), and change in depressive or anxiety symptoms. The primary outcome was a change in symptoms of anxiety or depression after a BT or BT+Ex intervention.

### 2.4. Bias and Limitations

A bias assessment was performed using the Cochrane Collaboration’s Tool for Assessing Risk of Bias [25]. Studies were evaluated for six domains of bias: selection, performance, detection, attrition, reporting, and other biases.

### 2.5. Statistical Analysis

Using a random effects model, a meta-analysis was conducted using Meta Essentials for Microsoft Excel [26]. For each reported depression and anxiety outcome, mean change was calculated as percent change from the baseline symptom score to account for variation in depression or anxiety scales between studies. Effect size (ES) for change in depression and anxiety symptoms was determined as the mean difference of BT and BT+Ex divided by pooled standard deviation. Studies were weighted using a random effects model to calculate a combined ES (Hedge’s g). Subgroup analyses were performed only if 10 studies were included for each outcome [27,28]. Targeted covariates within depression included exercise type (aerobic or combined aerobic and resistance exercise), exercise intensity (moderate or high intensity), and baseline depression level (elevated or non-elevated). Mind-body exercise was not included in covariate analysis of exercise type as there were too few studies. Additionally, no subgroup analyses could be performed within the anxiety studies due to the small number of studies. Meta-regression analyses were performed with total study duration (in weeks), percent of female subjects, and participant age as moderators. Statistical significance was set at *p* < 0.05.

## 3. Results

### 3.1. Literature Search and Publication Bias

**Figure 1:**
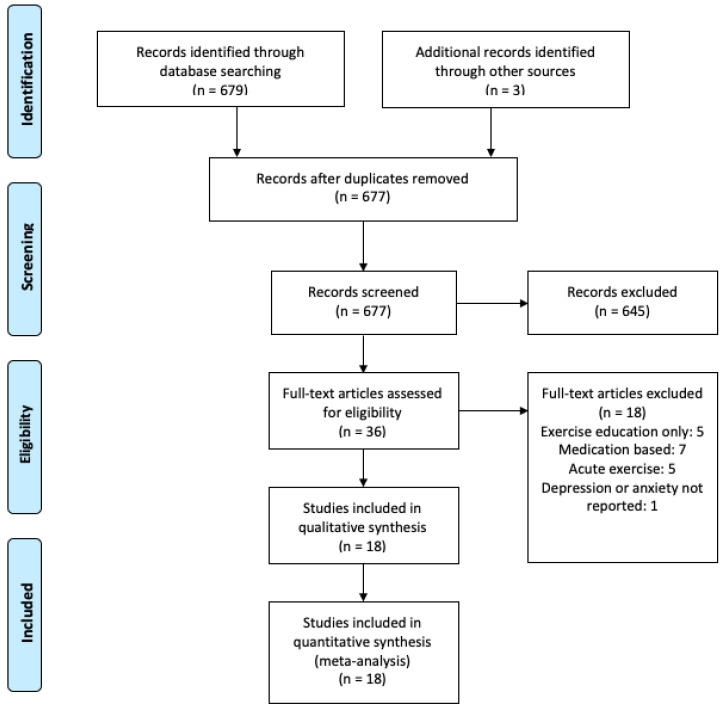
Process of publication selection.

The initial electronic searches yielded 679 records. A search of reference lists from retrieved review articles was manually reviewed and yielded an additional 3 records. After deletion of duplicates (n = 5), titles and abstracts were read to remove irrelevant records. A search in additional sources yielded 3 additional studies. An assessment of full text articles was performed for 36 articles; 18 studies met the inclusion criteria (Figure 1). All studies evaluated either depression, anxiety, or both using validated symptom scales. Study characteristics are presented in Table 1. Overall, the quality of studies was moderate to high. Randomization methods (random sequence generation) were reported in all studies. It was difficult to assess allocation concealment as details were not provided regarding the role of investigators enrolling patients. Participants and personnel were not blinded to the exercise study and outcomes, which indicates a high risk for bias; however, it is unlikely that this caused bias. All studies reported attrition, but evaluation of dropouts was not completed across all studies, and therefore the bias created is uncertain. All included studies reported clinically relevant outcomes.

### 3.2. Participants

Sample sizes of included studies varied from 23 to 785 participants (total = 1686, mean = 94), with an average age of 47.5 years (excluding subjects from non-BT or BT+Ex groups, i.e., control and exercise only). The majority of studies recruited both males and females with females comprising 65% of the study sample. Four studies recruited only female subjects [29,30,31,32]. A total of 12 RCTs reported comorbid conditions. Comorbidities included HIV [33], chronic obstructive pulmonary disease [34,35], heart failure [36], chronic low back pain [37,38], breast cancer [30], schizophrenia [22], binge eating disorder [31], PTSD [39], pregnancy [32] and stroke [40].

### 3.3. Intervention Characteristics

Behavioral therapy interventions included cognitive behavioral therapy (CBT), psychotherapy (Psy), stress management (SM), education (Edu), cognitive training, and a brief cognitive program classified as ‘‘other’’. The majority of studies employed CBT (n = 11) [29,31,34,36,38,40,41,42,43,44,45]. Two studies used mixed behavioral therapies (CBT+Psy [34], SM+Edu [35]). Nine studies employed aerobic exercise (AE) as the sole exercise type [22,31,32,33,36,41,42,44,45], two studies used resistance exercise (RE) [39,43], six studies combined AE and RE [30,34,35,37,38,40], and one study employed yoga [29]. No studies were identified that implemented Tai Chi exercise. Nine studies required participants to perform exercise in a supervised setting while two studies were home or gym based (not supervised by member of the research team).

Studies varied regarding the specific questionnaire used to assess depression and anxiety. Depression questionnaires included Beck Depression Inventory (BDI, n = 9), Center for Epidemiologic Studies Depression Scale (CES-D, n = 2), Hamilton Depression Rating Scale (HAM-D, n = 1), Hospital Anxiety and Depression Scale (HADS-D, n = 2), Taiwanese Depression Questionnaire (TDQ, n = 1), Depression Anxiety Stress Scales (DASS-D, n = 2), and Edinburgh Postnatal Depression Scale (EPDS, n = 1). Anxiety questionnaires included Hospital Anxiety and Depression Scale (HADS-A, n = 2), Depression Anxiety Stress Scales (DASS-A, n = 2), State-Trait Anxiety Inventory (STAI, n = 3) and Beck Anxiety Inventory (BAI, n = 1).

### 3.4. Effects of BT and BT+Ex on Depression

**Figure 2:**
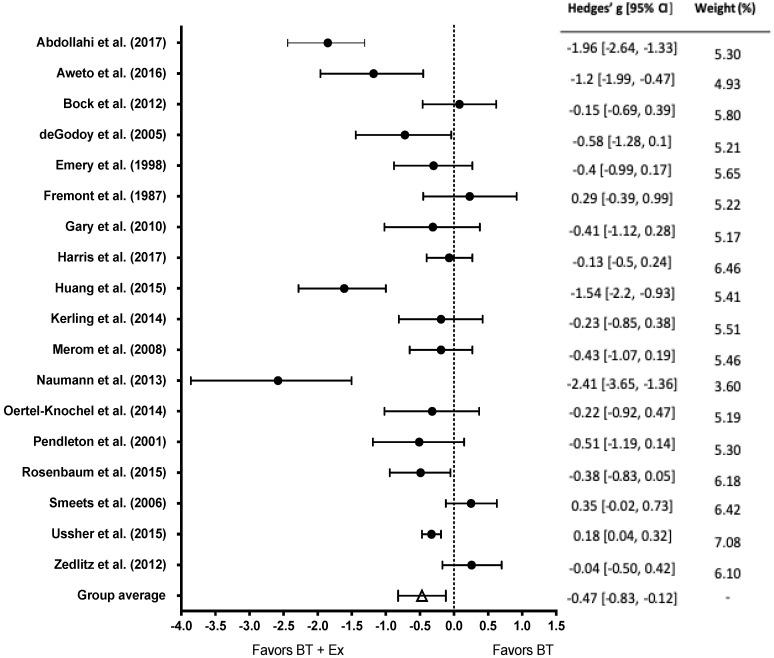
Overall effect of BT+Ex on depression. Test for overall effect g = −0.47, *p* = 0.005; BT+Ex = Behavioral Therapy + Exercise, BT = Behavioral Therapy only.

All studies examined the impact of BT (mean n of subjects = 46) vs. BT+Ex (mean n of subjects = 47) on depressive symptoms (n = 18). The effect of BT+Ex on depression was significant (g = −0.47; 95% CI [−0.83, −0.12]; *p* = 0.005), as displayed in Figure 2.

### 3.5. Subgroup Analysis of Exercise Intensity

**Figure 3:**
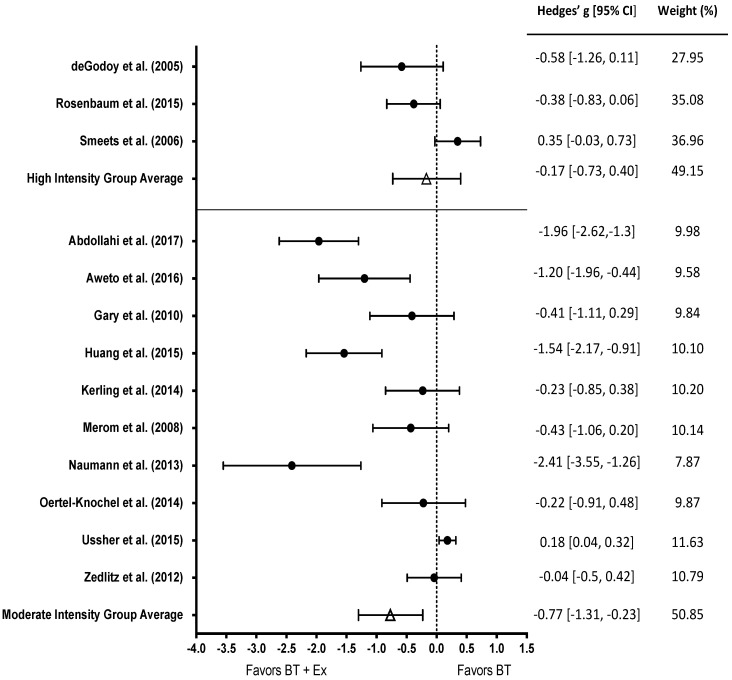
Subgroup analysis of BT+ high intensity (top) and BT+ moderate intensity exercise (bottom) among studies that measured depression. Test for overall effect of high intensity g = −0.17, *p* > 0.05. Test for overall effect of moderate intensity g = −0.77, *p* < 0.01. BT+Ex = Behavioral Therapy + Exercise, BT = Behavioral Therapy only.

In total, 13 studies reported exercise intensity and were included in the intensity subgroup analysis. Ten studies investigated the effect of moderate intensity exercise while three studies investigated the effect of high intensity exercise on depression (Figure 3). The effect of moderate intensity exercise was significant (g = −0.77, 95% CI [−1.31, −0.23]; *p* < 0.01); however, the effect of high intensity exercise interventions was not (g = −0.17, 95% CI [−0.73, −0.40]; *p* > 0.05).

### 3.6. Subgroup Analysis of Type of Exercise

**Figure 4:**
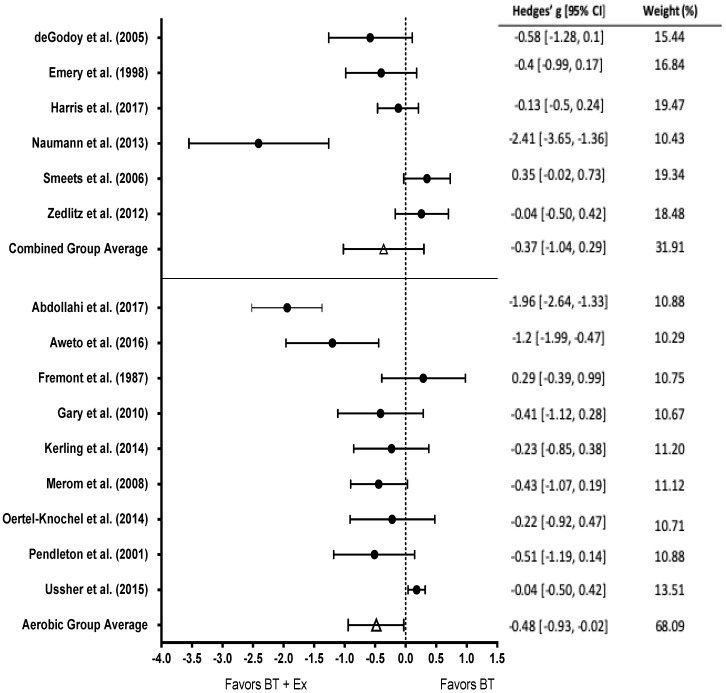
Subgroup analysis of combined aerobic and resistance (top) and aerobic exercise only (bottom) among studies that measured depression. Test for overall effect of combined exercise g = −0.37, *p* > 0.05. Test for overall effect of aerobic exercise g = −0.48, *p* < 0.05. BT+Ex = Behavioral Therapy + Exercise, BT = Behavioral Therapy only.

Nine studies employed an aerobic exercise intervention, six studies combined aerobic and resistance exercise (Figure 4). There was a significant effect for aerobic exercise (g = −0.48, 95% CI [−0.93, −0.02]; *p* = 0.03); however, there was no effect for combined exercise (g = −0.37, 95% CI [−1.04, 0.29]; *p* > 0.05).

### 3.7. Subgroup Analysis of Baseline Level of Depression

**Figure 5:**
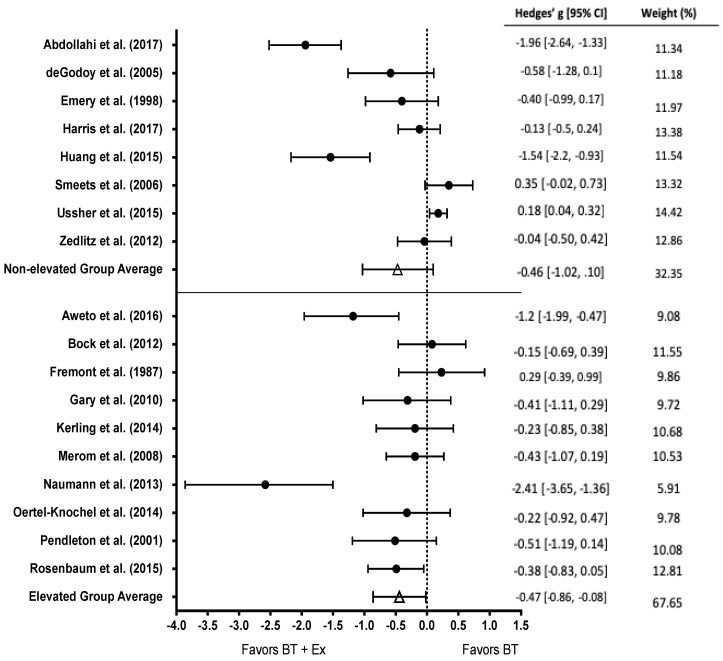
Subgroup analysis of non-elevated (top) and elevated baseline depression levels (bottom) among studies that measured depression. Test for overall effect of non-elevated baseline depression g = −0.46, *p* > 0.05. Test for overall effect of elevated baseline depression g = −0.47, *p* < 0.05. BT+Ex = Behavioral Therapy + Exercise, BT = Behavioral Therapy only.

Baseline level of depression was characterized according to the guidelines of each questionnaire used. Participants in nine studies were categorized as having elevated baseline depression levels, and nine studies recruited those with healthy baseline depression levels (Figure 5). There was a significant effect among those with elevated depression levels (g = −0.47, 95% CI [−0.86, −0.08]; *p* = 0.03); however, there was no effect for non-elevated baseline depression (g = −.46, 95% CI [−1.02, 0.10]; *p* > 0.05).

### 3.8. Depression Moderator Analysis

Average age of participants significantly moderated change in depression with an increase in age corresponding to a greater effect of BT+Ex on depression (lower depression scores) after the exercise intervention (*β* = −0.42; 95% CI [−0.05, 0.00]; *p =* 0.036). Percentage of female subjects (*β* = 0.14; 95% CI [−0.79, 1.53]; *p =* 0.498) and duration of intervention (*β* = 0.00; 95% CI [−0.12, 0.12]; *p =* 0.990) were not associated with effect size of BT+Ex.

### 3.9. Effects of BT and BT+Ex on Anxiety

**Figure 6:**
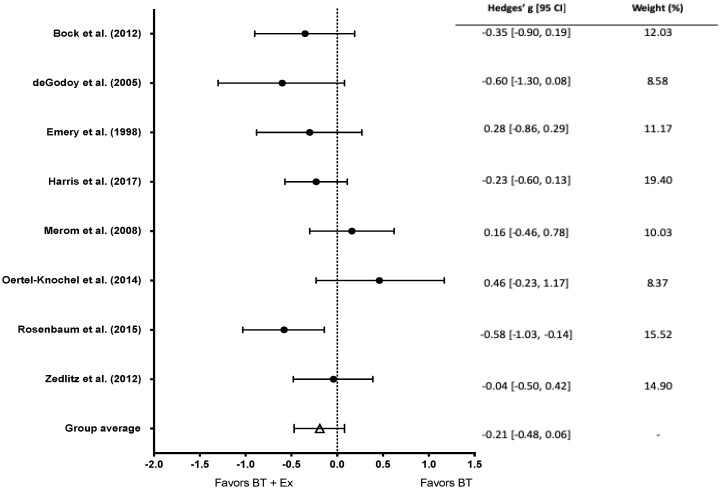
Overall effect of BT+Ex on anxiety. Test for overall effect g = −0.21, *p >* 0.05. BT+Ex = Behavioral Therapy + Exercise, BT = Behavioral Therapy only.

Eight studies reported pre- and post- anxiety scale scores. No significant effect of BT+Ex was observed for anxiety (Hedge’s g = −0.21, CI [−0.48 to 0.06]; *p =* 0.063, Figure 6).

### 3.10. Anxiety Moderator Analysis

Average age of participants (β = −0.46; 95% CI [−0.05, 0.02]; *p =* 0.237), percentage of female subjects (β = 0.46; 95% CI [−0.45, 1.58]; *p =* 0.189), and duration of intervention (β = −0.59; 95% CI [−0.17, 0.02]; *p =* 0.076) did not moderate the effect of BT+Ex on change in anxiety symptoms.

## 4. Discussion

The purpose of the present meta-analysis was to determine if the addition of exercise to BT results in augmented improvements of depression and anxiety symptoms. Results from this meta-analysis indicate that BT+Ex is a more effective treatment for depression than BT alone with a medium and significant negative effect size (Hedge’s g = −0.47) irrespective of exercise type, intensity, and baseline levels of depression. Further, findings indicate that this relationship is moderated by the average age of subjects with an increase in age corresponding to a greater effect of BT+Ex depression after the exercise intervention. Conversely, BT+Ex does not appear to be significantly more effective than BT for a reduction in anxiety (Hedge’s g = −0.21).

This meta-analysis compared the effectiveness of BT+Ex and BT alone on symptoms of depression and anxiety, when BT is defined as any type of behavioral therapy. A previous meta-analysis compared the effectiveness of CBT+Ex vs. CBT on change in depressive symptoms and found no significant difference between the two intervention groups (CBT+Ex vs. CBT alone) [20]. It is also important to note that the authors included studies that used both exercise interventions and exercise education interventions. The inclusion of studies lacking an active exercise component may explain the differing results with the present study. Further, Bernard et al. [20] included studies that recruited only participants with chronic disease. The inclusion of both healthy and clinical population studies in the present meta-analysis may further explain the differences observed in the current results. For example, BT+Ex had a large effect (g = −2.41) on lowering depression among breast cancer survivors [30]. This population, similar to others, is susceptible to other comorbidities, such as obesity, and low cardiovascular fitness. Exercise may assist with treating these additional abnormalities, which likely improves one’s behavioral state. Interpretations should be made with caution as the sample size, and thus the weight of the included study was small [30].

Depression is characterized by both behavioral and physiological pathologies. The psychobiological nature of depression may, in part, explain why BT+Ex was shown to be effective compared to BT in the present study. Specifically, the therapeutic benefit of psychotherapy is largely attributed to an improvement in psychological constructs, while the therapeutic benefit of exercise may be due to physiological changes. Regarding psychological constructs, depression is related to dysfunctional attitudes, negative thoughts, and rumination. Psychotherapy is proposed to reduce the prevalence of these components, thereby yielding an antidepressant effect through psychological changes [46]. Alternately, depression has been associated with reduced size of brain regions that play a role in emotion regulation including the hippocampus, anterior cingulate cortex, and prefrontal cortex [47]. Interestingly, exercise intervention studies have reported structural changes and increased size of these brain regions in response to the intervention [48,49]. Researchers speculate that the volumetric changes are a result of increased neurogenesis due to exercise-induced expression of neurotrophic factors [50]. Further, the neural benefits of exercise may be augmented in individuals experiencing age-related brain atrophy, which potentially explains the moderating effect of age reported in the present study [50]. The psychological and physiological changes promoted through BT+Ex may explain the greater effect of the combined treatment compared to BT alone.

In contrast to depression, BT+Ex was not significantly more effective than BT alone for reducing symptoms of anxiety. This may be due to a more immediate role of exercise in reducing anxiety. One proposed mechanism regarding the anxiolytic effect of exercise is termed the ‘‘endorphin hypothesis’’. This theory speculates that the acute release of β-endorphins reduces anxiety symptoms, thus explaining the role of exercise on anxiety [51]. Thus, the transient nature of the therapeutic benefit of exercise may explain why no differences were observed between BT+Ex and BT alone, as the anxiolytic effects were not observed after acute exercise, but rather after a chronic exercise intervention.

### Limitations

Despite the moderate to high quality of the included studies, there are several limitations that should be noted. First, several studies did not report intensity of exercise (n = 6), potentially masking or skewing the intensity-dependent effect of exercise on depression and anxiety. Second, only 13 of the studies used a sample population with elevated depressive symptoms, which may temper the effect of the BT and BT+Ex intervention. Third, studies used various diagnostic criteria for depression and anxiety, limiting the ability to directly compare the magnitude of effect across all studies. Fourth, high levels of statistical heterogeneity were observed within depression analyses among studies. This may be explained by variations exercise program characteristics (e.g., exercise intensity, type), behavioral therapy characteristics (e.g., type of therapy), and overall intervention characteristics (e.g., duration, group vs. individualized, reason for treatment). Future studies should provide inclusive methodological descriptions of the exercise intervention (type, duration, intensity), recruit individuals with elevated levels of depression or anxiety, measure depression and anxiety using common methods of assessment (i.e., BDI, HADS).

## 5. Conclusions

The findings of this meta-analysis provide support for the use of exercise as an effective adjunct treatment for depression across a range of comorbidities. Specifically, the addition of moderate intensity exercise to BT may yield superior improvements in depression symptoms. Similarly, aerobic exercise interventions appear to be more beneficial than combined aerobic plus resistance training programs. Further, the moderating effect of age on BT+Ex indicates that the effectiveness of BT+Ex may be augmented in older individuals. Together, exercise may provide an inexpensive, effective addition to traditional behavioral therapy. To develop individualized BT+Ex programs, future research is needed to elucidate the mechanistic changes that explain the benefits of BT+Ex for depression and anxiety reduction.

## Figures and Tables

**Table 1:** Study Characteristics.

Author	Year	Behavioral Therapy						Exercise Therapy				Disorder	Diagnostic Criteria	Comorbidity
		CBT	Psy	SM	Edu	Other		AE	RE	Yoga				
Abdollahi	2017	•	◦	◦	◦	◦	Group CBT, 90 min, 1×/week, 12 weeks	•	◦	◦	Supervised moderate intensity, 35 min, 3×/week, 12 weeks	Depression	BDI-II	None
Aweto	2016	◦	•	◦	◦	◦	Group counselling, 30 min, 1×/2 weeks, 6 weeks	•	◦	◦	Supervised, moderate intensity, 30 min, 3×/week, 6 weeks	Depression	BDI	HIV
Bock	2012	•	◦	◦	◦	◦	Group CBT for smoking cessation, 60 min, 1×/week, 8 weeks	◦	◦	•	Supervised vinyasa yoga, 60 min, 2×/week, 8 weeks	Depression, Anxiety	CES-D, STAI	None
De Godoy	2005	•	•	◦	◦	◦	Individual psychotherapy and CBT, 1×/week, 12 weeks	•	•	◦	Supervised, moderate to high intensity, 2×/week, 12 weeks	Depression, Anxiety	BDI, BAI	COPD
Emery	1998	◦	◦	•	•	◦	Group SM, 60 min, 4×/week, 5 weeks followed by 60 min, 1×/week, 5 weeks	•	•	◦	Group exercise, 45 min, 5×/week, 5 weeks followed by 60-90 min, 3×/week, 5 weeks	Depression, Anxiety	CES-D, STAI	COPD
Fremont	1987	•	◦	◦	◦	◦	Individual CBT, 60 min, 1×/week, 10 weeks	•	◦	◦	Supervised group exercise, 20 min, 3×/week, 10 weeks	Depression	BDI	None
Gary	2010	•	◦	◦	◦	◦	Individual, home-based CBT, 30-45 min, 1×/week, 12 weeks	•	◦	◦	Home-based exercise, 60 min, 3×/week, 12 weeks	Depression	HAM-D	Heart Failure
Harris	2017	◦	◦	◦	◦	•	Individual, brief cognitive program, 2-4 sessions, 5-day period	•	•	◦	Supervised, individualized exercise 90 min, 3×/week, 12 weeks	Depression, Anxiety	HADS	Chronic low back pain
Huang	2015	•	◦	◦	◦	◦	Group CBT, 20-25 min, 1×/week, 8 weeks	◦	•	◦	Supervised exercise, 30 min, 2×/week, 8 weeks	Depression	TDQ	None
Kerling	2014	•	◦	◦	◦	◦	Unspecified CBT	•	◦	◦	Supervised exercise, 45 min, 3×/week, 6 weeks	Depression	BDI	None
Merom	2008	•	◦	◦	◦	◦	Group CBT, 90 min, 1×/week, 10 weeks	•	◦	◦	Supervised group exercise, 30 min, 5×/week, 10 weeks	Depression, Anxiety	DASS	None
Naumann	2013	◦	•	◦	◦	◦	Individual counselling, 60 min, 1×/week, 8 weeks	•	•	◦	Supervised exercise, 45-60 min, 3×/week, 8 weeks	Depression	BDI	Breast cancer survivors
Oertel-Knochel	2014	◦	◦	◦	◦	•	Group cognitive training, 30 min, 3×/week, 12 weeks	•	◦	◦	Supervised exercise, 45 min, 3×/week, 4 weeks	Depression, Anxiety	BDI-II, STAI	Schizophrenia
Pendleton	2010	•	◦	◦	◦	◦	Individual CBT, 90 min, 1×/week, 16 weeks	•	◦	◦	Unsupervised exercise, 45 min, 3×/week, 16 weeks	Depression	BDI-II	Binge eating disorder
Rosenbaum	2015	◦	•	◦	◦	◦	Psychotherapy and group therapy	◦	•	◦	Supervised exercise, 30 min, 3×/week, 12 weeks	Depression, Anxiety	DASS	Post-traumatic stress disorder
Smeets	2006	•	◦	◦	◦	◦	Individual CBT, 11.5 h total, 10 weeks	•	•	◦	Supervised exercise, 105 min, 3×/week, 10 weeks	Depression	BDI-II	Chronic low back pain
Ussher	2015	◦	◦	◦	◦	•	Individual behavioral support, 20 min, 1×/week, 6 weeks	•	◦	◦	Supervised, moderate intensity exercise, 30 min, 2×/week, 6 weeks	Depression	EPDS	Pregnancy
Zedlitz	2012	•	◦	◦	◦	◦	Group CBT, 120 min, 2×/week, 12 weeks	•	•	◦	Supervised exercise, 120 min, 2×/week, 12 weeks	Depression, Anxiety	HADS	Stroke

CBT = cognitive behavioral therapy, Psy = psychotherapy, SM = stress management, Edu = education, AE = aerobic exercise, RE = resistance exercise, BDI = Beck Depression Inventory, CES-D = Center for Epidemiologic Studies Depression Scale, HAM-D = Hamilton Depression Rating Scale, HADS = Hospital Anxiety and Depression Scale, TDQ = Taiwanese Depression Questionnaire, DASS-D = Depression Anxiety Stress Scales, EPDS = Edinburgh Postnatal Depression Scale, STAI = State-Trait Anxiety Inventory, BAI = Beck Anxiety Inventory, black circles (•) = intervention used, clear circles (◦) = intervention not used.
